# Pramipexole in patients with early Parkinson's disease (PROUD): a randomised delayed-start trial

**DOI:** 10.1016/S1474-4422(13)70117-0

**Published:** 2013-08

**Authors:** Anthony HV Schapira, Michael P McDermott, Paolo Barone, Cynthia L Comella, Stefan Albrecht, Helen H Hsu, Daniel H Massey, Yoshikuni Mizuno, Werner Poewe, Olivier Rascol, Kenneth Marek

**Affiliations:** a Department of Clinical Neurosciences, University College London Institute of Neurology, London, UK; b University of Rochester Medical Center, Rochester, NY, USA; c University of Salerno, Italy; d Department of Neurological Sciences, Rush University Medical Center, Chicago, Illinois, USA; e Boehringer Ingelheim GmbH, Ingelheim, Germany; f Boehringer Ingelheim Pharmaceutical Inc, Ridgefield, CT, USA; g Boehringer Ingelheim Ltd, Bracknell, Berkshire, UK; h Department of Neuroregenerative Medicine, Kitasato University School of Medicine, Minami-ku, Sagamihara, Kanagawa, Japan; i Department of Neurology, Medical University Innsbruck, Innsbruck, Austria; j Departments of Clinical Pharmacology and Neurosciences, University Hospital of Toulouse and Clinical Investigation Centre INSERM CIC9302 and UMR825, University of Toulouse III, Toulouse, France; k Institute for Neurodegenerative Disorders, New Haven, CT, USA

## Abstract

**Background:**

In models of dopaminergic neuronal loss, the dopamine agonist pramipexole has exhibited neuroprotective properties. The Pramipexole On Underlying Disease (PROUD) study was designed to identify whether early versus delayed pramipexole initiation has clinical and neuroimaging benefits in patients with Parkinson's disease (PD).

**Methods:**

Between May 24, 2006, and April 22, 2009, at 98 centres, we recruited patients with PD diagnosed within 2 years and aged 30–79 years. We randomly assigned eligible patients (ratio 1:1), by a centralised, computerised randomisation schedule, to receive double-blind either placebo or pramipexole (1·5 mg a day) and followed them up for 15 months. At 9 months, or as early as 6 months if considered necessary, placebo recipients were assigned to pramipexole. In a neuroimaging substudy, striatal dopamine-transporter binding was assessed by SPECT. All patients, investigators, and independent raters were masked to study treatment. The primary endpoint was the 15-month change from baseline in total score on the unified Parkinson's disease rating scale (UPDRS). This trial is registered with ClinicalTrials.gov, number NCT00321854.

**Findings:**

Of 535 patients, 261 were randomly assigned to receive pramipexole and 274 to receive placebo. At 15 months (n=411), adjusted mean change in UPDRS total score showed no significant difference between early and delayed pramipexole (−0·4 points, 95% CI −2·2 to 1·4, p=0·65). 62 patients in the early pramipexole group and 61 patients in the delayed pramipexole group were included in the neuroimaging substudy, for which the adjusted mean 15-month change in striatal ^123^I-FP-CIT binding was −15·1% (SE 2·1) for early and −14·6% (2·0) for delayed pramipexole (difference −0·5 percentage points, 95% CI −5·4 to 4·4, p=0·84). Overall, 180 (81%) of patients given early pramipexole and 179 (84%) patients given delayed pramipexole reported adverse events (most frequently nausea), and 22 (10%) patients in the early pramipexole group and 17 (8%) in the delayed pramipexole group had serious events, two of which (hallucinations and orthostatic hypotension) were deemed related to study drug.

**Interpretation:**

By clinical and neuroimaging measures, pramipexole showed little evidence differentiating 15-month usage from usage delayed for 6–9 months. The results do not support the hypothesis that pramipexole has disease-modifying effects.

**Funding:**

Boehringer Ingelheim GmbH.

## Introduction

Parkinson's disease (PD) is a progressive neurodegenerative disorder in which loss of dopaminergic neurons of the substantia nigra pars compacta underlies the major early motor features by which the disease is diagnosed clinically. Although several therapeutic strategies are available to treat the dopamine deficiency of PD and have been shown to improve motor symptoms, no drug has yet been shown unequivocally to slow the progression of the loss of dopamine cells.^1^, ^2^ Development of a therapy to slow progression of neurodegeneration in PD is a major unmet need.

Pramipexole is a dopamine D2/D3 receptor agonist with proven efficacy in the treatment of PD motor symptoms in early and advanced PD.^3^ In cell culture studies^4^ and studies in rodents^5^ and primates,^6^ pramipexole showed neuroprotective properties that seemed to arise partly by a mitochondria-mediated anti-apoptotic mechanism. These results were the basis for considering that, in addition to its symptomatic action, pramipexole might have a disease-modifying effect.

The Pramipexole On Underlying Disease (PROUD) study was designed to identify whether early, as opposed to delayed, initiation of pramipexole resulted in improved outcome, as defined by unified Parkinson's disease rating scale (UPDRS) total score.^7^ We undertook a neuroimaging substudy to assess the effect of the drug on the rate of loss of dopamine transporter binding.^8^ PROUD is the second prospectively designed delayed-start trial in PD, and to our knowledge the first to combine clinical and neuroimaging endpoints.

## Methods

### Patients and study design

PROUD is a randomised, double-blind, placebo-controlled, delayed-start trial of pramipexole in patients with early PD. The clinical trial design of PROUD has been published previously and the reader is referred to the report for detailed discussion of the design.^7^

We recruited patients at 98 centres in ten countries (Austria, Finland, France, Germany, Italy, Japan, Spain, Sweden, the UK, and the USA). Patients were 30–79 years (extended from 75 years by protocol amendment in October 2006, to facilitate enrolment), had idiopathic PD characterised by bradykinesia plus at least two further PD signs (resting tremor, rigidity, or asymmetry), were at modified Hoehn and Yahr^9^ stage 1 or 2, were diagnosed within the preceding 2 years, and were judged unlikely to need symptomatic treatment for at least the next 6 months, preferably 9 months. We excluded potential patients if they were currently using PD drugs, had used antipsychotic drugs within the preceding 6 months, or had any clinically significant abnormalities unrelated to PD in physical findings or laboratory values; we also excluded patients with medical or psychiatric disorders capable of interfering with study participation or the interpretation of study data, and those with any history of psychosis, dementia, or major or seasonal depression.

The study was conducted in accordance with its protocol, with good clinical practice, and with the provisions of the Declaration of Helsinki and its amendments. Before patients were enrolled, the protocol, the informed-consent form, and all protocol amendments were approved by local Institutional Review Boards or Independent Ethics Committees. The nature and purpose of the study were explained to all patients, who provided written informed consent before any study procedures.

### Randomisation and masking

Patients were randomly assigned (1:1 ratio) by centralised, computerised, sponsor-maintained randomisation schedule to receive double-blind pramipexole or placebo.

Study drug (ie, pramipexole tablets or matching placebo) was up-titrated over 4 weeks from 0·125 mg three times a day to 0·25 mg three times a day, and finally 0·5 mg three times a day, a fixed maintenance level shown to provide adequate symptomatic benefit and intended to minimise withdrawal due to adverse events in those assigned to active drug.

At 9 months, or as early as 6 months if an investigator elected it for a patient expressing inability to tolerate PD symptoms, participants were all assigned to the pramipexole regimen, including its double-blind up-titration. For all patients, the month 9 visit (which could be conducted as much as 3 months earlier) marked the transition from study period 1 (double-blind pramipexole *vs* placebo) to period 2 (double-blind early *vs* delayed pramipexole). Any patient needing additional PD treatment discontinued the study. Non-PD treatment (including anti-emetics) was permitted for the welfare of the patient, on the basis of investigator judgment.

All patients and investigators were masked to study treatment. Pramipexole tablets and matching placebo were given in identical blister packaging. Masking was maintained during period 2 for all but two patients unmasked for non-emergencies. An independent masked rater distinct from the study investigators assessed patients at baseline and 15 months, and was separate from the masked investigator who assessed patients at every visit.

### Procedures

Independent raters assessed the 15-month change from baseline in total score on the UPDRS (the sum of part I [behaviour, mentation, and mood], part II [activities of daily living], and part III [motor function]). A study investigator assessed the UPDRS at 3 months, 6 months, 9 months, and 15 months. The independent rates also assessed the clinical global impression-global improvement (CGI-I) and clinical global impression-severity of illness (CGI-S) scales^10^ applied at 15 months. Additionally, at 6–9 months (ie, end of period 1) and at 15 months (end of period 2), patients assessed their quality of life on the Parkinson's disease questionnaire (PDQ-39), the EuroQoL five-dimension questionnaire (EQ-5D), and the EuroQoL visual analogue scale (EQVAS). Patients also completed the Beck depression inventory version 1A (BDI).

The imaging substudy group was a subset of patients in the PROUD study who provided additional consent for this substudy and who had a baseline and a post-baseline (15 months) assessment of striatal dopamine transporter density, as measured by SPECT at sites with tracer availability.^8^ Striatal binding of ^123^I-FP-CIT, a marker of dopamine-transporter density, was assessed by standardised SPECT and interpreted centrally by one of the lead investigators (KM).

We assessed safety and tolerability descriptively from the incidence, types, and severity of reported adverse events and the incidence of withdrawal due to adverse events. At months 1, 6, 9, 12, and 15, we assessed the incidence of impulse control disorders by the modified Minnesota impulsive disorders interview (mMIDI),^11^ consisting of items on gambling, compulsive buying, and compulsive sexual behaviour.

### Statistical analyses

The primary outcome variable was the 15-month change from baseline in total score on the UPDRS, as assessed by an independent rater. Secondary assessments included the UPDRS assessed at 3, 6, 9, and 15 months by a study investigator and the CGI-I and CGI-S applied at 15 months by the independent raters.

The period 2 full-analysis set included all patients randomly assigned to treatment who took at least one dose of study drug and had UPDRS scores assessed by an independent rater at baseline and at least once during period 2. In this set, we compared mean 15-month change in total UPDRS score between patients assigned to early pramipexole versus delayed pramipexole using analysis of covariance (ANCOVA), with country and baseline score as covariates. We expected the final UPDRS total score at month 15 to be correlated with the baseline score. Therefore, we selected an ANCOVA model for the primary analysis change in UPDRS total score from baseline at the end of the second maintenance phase (ie, period 2; month 15) with factors treatment and centre and the covariate UPDRS total score at baseline. For patients who discontinued prematurely during period 2, we used scores at their time of withdrawal for analysis.

On the basis of results from a previous trial,^12^ we did a post-hoc analysis of the change in 15-month total score separately in patients with baseline total scores of 25 or higher and in those with total scores lower than 25. We also analysed other UPDRS score changes by ANCOVA including the slope of total-score change from month 3 to month 9 in all patients with at least two UPDRS assessments during period 1. We calculated the slope as the difference between the last and first UPDRS observations during this period divided by the number of weeks between these observations. We analysed changes in quality of life ratings using Wilcoxon rank sum tests with treatment effects summarised by Hodges-Lehmann estimates. We analysed BDI changes as the sum of 21 item scores. We collapsed CGI-I ratings into three categories—much or very much improved, essentially unchanged, and much or very much worse—and analysed these groups for odds of category improvement using multinomial logistic regression (with terms for treatment, country, and CGI-S). On CGI-S, we defined changes of one rating point or fewer as essentially unchanged.

In the neuroimaging substudy, we analysed mean binding change by ANCOVA (with adjustment for centre and baseline value). We imputed missing data using last observation carried forward, although we accept that this method has limitations.^13^

On the basis of previous data for pramipexole in early PD clinical trials,^14^, ^15^ using nQuery Advisor Release 4.0 (Statistical Solutions), we calculated a sample size of 190 participants per group to provide 80% power to detect a three-point group difference in mean 15-month change in UPDRS total score, assuming SD 10·4 points and a significance level of 5% (two-tailed).^7^ To compensate for the anticipated 24% rate of withdrawal during period 1, we increased the sample size to 250 participants per group (500 participants in total). This trial is registered with ClinicalTrials.gov, number NCT00321854.

### Role of the funding source

The sponsor of the study, together with the independent lead investigators (independent authors of this report) had a role in study design, data collection, data analysis, and data interpretation, and also supported the reporting of study results, partly as employer of some of the authors. AHVS drafted the report. AHVS and all authors had full access to all the data and contributed to the revision of the report. The corresponding author, AHVS, had final responsibility for the decision to submit for publication.

## Results

**Figure 1:**
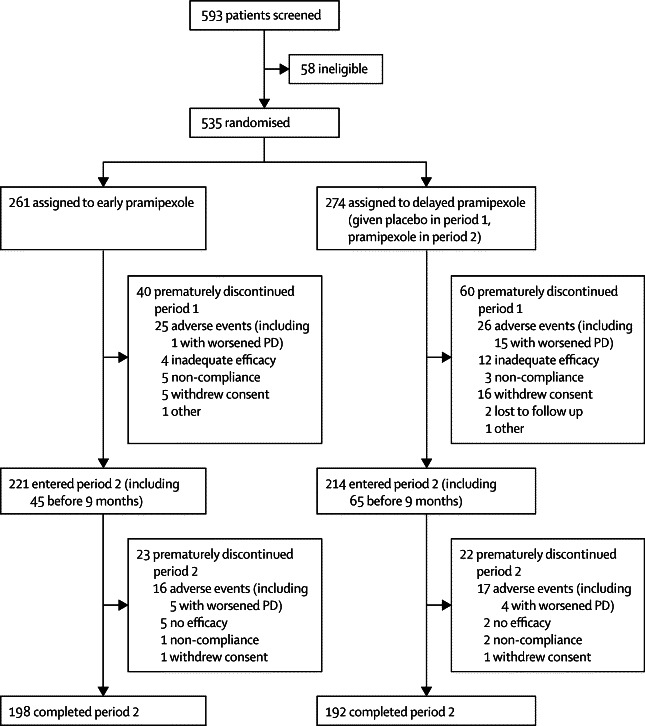
Trial profile PD=Parkinson's disease.

We recruited patients between May 24, 2006, and April 22, 2009. Of 593 patients screened, 535 were randomly assigned to either early pramipexole (261 patients) or delayed pramipexole (274 patients; figure 1). 221 (85%) of 261 patients assigned to early pramipexole and 214 (78%) of 274 patients assigned to delayed pramipexole (ie, placebo) completed period 1 of the study. Of patients who entered period 2, 45 (20%) of 221 patients who received early pramipexole did so before 9 months, compared with 65 (30%) of 214 patients who received delayed pramipexole. Of the 435 patients entering period 2, 198 (90%) of the patients in the early pramipexole group (or 76% of the initial group) and 192 (90%) of the patients in the delayed pramipexole group (or 70% of the initial group) completed period 2. In both treatment groups during both periods, adverse events were the main reason for premature discontinuation (figure 1). The most common adverse events leading to premature discontinuation in the delayed group during period 1 were related to need for symptomatic PD treatment. Overall, mean compliance with treatment (expressed as percentage of prescribed doses taken) was 95·9% (SD 10·3) in the early pramipexole group and 95·3% (12·5) in the delayed pramipexole group.

The groups were similar in terms of demographic and clinical characteristics at baseline (table 1). In 195 (75%) of 261 patients assigned to early pramipexole and 199 (73%) of 274 patients assigned to delayed pramipexole, PD had been diagnosed 6 months or less before baseline.

**Table 1:** Baseline characteristics of patients

	**All patients**		**Period 2 full-analysis set**	
	Early pramipexole group (n=261)	Delayed pramipexole group (n=274)	Early pramipexole group (n=211)	Delayed pramipexole group (n=200)
**Age (years)**				
Mean (SD)	62·1 (10·1)	62·9 (9·9)	62·0 (9·9)	62·2 (10·0)
Median (IQR)	64·0 (56·0–70·0)	64·0 (57·0–70·0)	64·0 (57·0–69·0)	63·0 (55·5–70·0)
**Sex**				
Male	177 (68%)	166 (61%)	146 (69%)	120 (60%)
Female	84 (32%)	108 (39%)	65 (31%)	80 (40%)
**Race**				
White	251 (96%)	261 (95%)	201 (95%)	189 (94%)
Asian	9 (3%)	10 (4%)	9 (4%)	9 (4%)
Black	1 (<1%)	3 (1%)	1 (<1%)	2 (1%)
**PD duration (months)**				
Mean (SD)	4·4 (6·3)	4·5 (5·9)	4·5 (5·5)	4·8 (6·1)
Median (IQR)	1·8 (0·6–6·1)	1·7 (0·5–6·6)	1·9 (0·7–6·7)	2·0 (0·5–7·1)
**Hoehn and Yahr stage**				
1·0	93 (36%)	120 (4%)	73 (35%)	88 (44%)
1·5	59 (23%)	50 (18%)	54 (26%)	39 (20%)
2·0	109 (4%)	104 (38%)	84 (40%)	73 (36%)
**UPDRS total score**				
By independent rater	24·2 (10·5)*; 23·0 (16·0–31·0)*	25·0 (11·2); 24·0 (16·0–32·0)	24·2 (10·4); 23·0 (16·0–30·0)	24·1 (10·3); 23·0 (16·0–30·0)
By study investigator	23·7 (10·0)†; 22·0 (17·0–29·0)†	24·1 (10·7)‡; 23·0 (16·0–32·0)‡	23·3 (9·6)§; 22·0 (17·0–29·0)§	23·3 (10·1); 21·0 (16·0–30·0)
**Quality of life ratings**				
PDQ-39 total score	10·0 (4·7–17·0)†	9·4 (5·1–17·9)‡	9·8 (4·5–15·5)	9·3 (4·8–16·5)
EQ-5D total score	0·78 (0·69–1·00)	0·78 (0·69–1·00)‡	0·78 (0·69–1·00)	0·78 (0·69–1·00)
EQVAS	80 (70–87)	80 (70–90)	80 (70–88)	80 (70–90)
**BDI total score**				
Mean (SD)	6·4 (5·5)	6·7 (5·7)‡	6·1 (5·4)	6·3 (5·6)¶
Median (IQR)	5·0 (2·0–9·0)	5·0 (2·0–10·0)‡	5·0 (2·0–9·0)	5·0 (2·0–9·0)¶

All data are mean (SD), median (IQR), or number of patients (%). BDI=Beck depression inventory. EQ-5D=EuroQoL five-dimension questionnaire. EQVAS=EuroQoL visual analogue scale. PD=Parkinson's disease. PDQ-39=39-item Parkinson's disease questionnaire. UPDRS total score=Unified Parkinson's disease rating scale, sum of parts I, II, and III.

* n=259.

† n=260.

‡ n=273.

§ n=210.

¶ n=199.

For period 1, recipients of early pramipexole showed relative stability in total UPDRS scores: in the period 2 full-analysis set, the adjusted mean change over the first 6–9 months of the study was −0·5 (SE 0·6) points, as assessed by study investigators, compared with 4·3 (0·6) for placebo recipients, a difference of −4·8 points (95% CI −6·3 to −3·2, p<0·0001; table 2). In months 3–9, the adjusted mean slope of the total score change in the period 1 full-analysis set was 0·11 (SE 0·04) points per week in the early pramipexole group, compared with 0·22 (0·04) for delayed pramipexole group, a difference of −0·10 (95% CI −0·19 to −0·01, p=0·03). At 9 months, all three quality of life scales and the BDI exhibited significant differences favouring pramipexole (table 3).

**Table 2:** Adjusted mean changes on UPDRS part I, part II, part III, and total score (period 2 full-analysis set)

		**Adjusted mean change (SE)**		**Difference (95% CI); p value**†
		Early pramipexole group (n=210 or 211)*	Delayed pramipexole group (n=200)	
**UPDRS total score**‡				
Independent rater				
	15 months	0·3 (0·7)	0·7 (0·7)	−0·4 (−2·2 to 1·4); 0·65
Study investigator				
	9 months	−0·5 (0·6)	4·3 (0·6)	−4·8 (−6·3 to −3·2); <0·0001
	15 months	0·6 (0·7)	0·5 (0·7)	0·0 (−1·7 to 1·8); 0·96
**UPDRS part I**				
Independent rater				
	15 months	−0·3 (0·1)	0·0 (0·1)	−0·3 (−0·5 to 0·0); 0·04
Study investigator				
	9 months	−0·2 (0·1)	0·1 (0·1)	−0·3 (−0·5 to −0·1); 0·02
	15 months	−0·2 (0·1)	−0·1 (0·1)	0·2 (−0·4 to 0·1); 0·16
**UPDRS part II**				
Independent rater				
	15 months	0·5 (0·2)	0·4 (0·2)	0·0 (−0·6 to 0·6); 0·93
Study investigator				
	9 months	0·4 (0·2)	1·5 (0·2)	−1·1 (−1·7 to −0·5); 0·0001
	15 months	0·6 (0·2)	0·6 (0·2)	0·0 (−0·6 to 0·6); 0·98
**UPDRS part III**				
Independent rater				
	15 months	0·1 (0·5)	0·3 (0·5)	−0·2 (−1·5 to 1·1); 0·80
Study investigator				
	9 months	−0·6 (0·5)	2·7 (0·5)	−3·3 (−4·5 to −2·2); <0·0001
	15 months	0·2 (0·5)	−0·1 (0·5)	0·2 (−1·1 to 1·5); 0·74

ANCOVA=analysis of covariance. UPDRS=unified Parkinson's disease rating scale.

* Depending on timepoint.

† ANCOVA adjusted for country and baseline.

‡ Total of parts I, II, and III.

**Table 3:** Changes on quality of life scales and BDI by timepoint (period 2 full-analysis set)

	Early pramipexole group (n=208–211)*	Delayed pramipexole group (n=197–200)*	**Difference (95% CI)**†**; p value**‡
**PDQ-39 total score**			
9 months	−0·5 (−3·6 to 2·0)	1·4 (−2·2 to 5·0)	−2·0 (−3·1 to −0·9); 0·0001
15 months	−0·4 (−3·2 to 3·8)	0·3 (−3·6 to 4·4)	−0·6 (−1·8 to 0·7); 0·215
**EQ-5D total score**			
9 months	0·00 (−0·03 to 0·09)	0·00 (−0·14 to 0·00)	0·05 (0·00 to 0·09); <0·0001
15 months	0·00 (−0·03 to 0·09)	0·00 (−0·08 to 0·08)	0·00 (0·00 to 0·03); 0·261
**EQVAS**			
9 months	0·0 (−5·5 to 5·0)	−0·5 (−10·0 to 5·0)	3·0 (0·0 to 5·0); 0·028
15 months	0·0 (−8·0 to 7·0)	0·0 (−10·0 to 2·0)	2·0 (0·0 to 5·0); 0·049
**BDI, adjusted**†			
9 months	−1·1 (0·3)	0·3 (0·3)	−1·4 (−2·2 to −0·6); 0·0009
15 months	−1·0 (0·3)	−0·5 (0·3)	−0·5 (−1·3 to 0·2); 0·1702

Data are median change (IQR) or mean change (SE), unless otherwise indicated. ANCOVA=analysis of covariance. BDI=Beck depression inventory. EQ-5D=EuroQoL five-dimension questionnaire. EQVAS=EuroQoL visual analogue scale. PDQ-39=39-item Parkinson's disease questionnaire.

* Depending on timepoint.

† For quality of life measures, Hodges-Lehmann estimate. For BDI, adjusted for baseline and country.

‡ For quality of life measures, Wilcoxon rank sum test, stratified by country. For BDI, ANCOVA adjusted for baseline and country.

**Figure 2:**
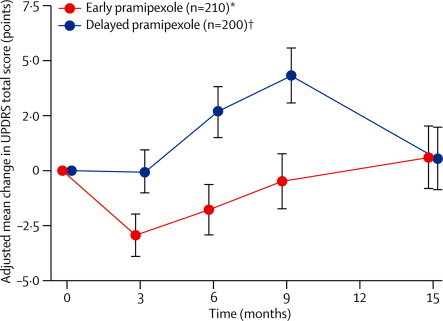
15-month time course of UPDRS total score (study investigators' ratings; period 2 full-analysis set) UPDRS total score was the sum of parts I. II, and III; means were adjusted for country and baseline. 95% CIs are shown at every timepoint. UPDRS=unified Parkinson's disease rating scale. *n=210 at all timepoints, one patient was excluded because of missing baseline data. †n=200 at all timepoints with the exception of month 3 (n=198).

In period 2, the adjusted mean change in UPDRS total score at 15 months as assessed by independent raters (the primary outcome variable), was 0·3 (SE 0·7) points in the early pramipexole group and 0·7 (0·7) points in the delayed pramipexole group, a difference of −0·4 (95% CI −2·2 to 1·4, p=0·65). At endpoint, the adjusted mean total scores were 24·5 (SE 0·7) in the early pramipexole group and 24·9 (0·7) in the delayed pramipexole group. For ratings by study investigators, figure 2 shows the 15-month time course of the adjusted mean total score change in each treatment group. The adjusted mean 15-month change was 0·6 (SE 0·7) points in the early pramipexole group and 0·5 (0·7) points in the delayed pramipexole group (95% CI −1·7 to 1·8, p=0·96). Findings for UPDRS part I, part II, part III, and total score are provided in table 2.

At 15 months, two of the three quality of life measures and the mean BDI score did not differ significantly between treatment groups. The third quality of life scale, the EQVAS, continued to show a small difference that favoured early pramipexole but was of doubtful clinical significance (table 3). At 15 months, the odds ratio for CGI-I category improvement was 0·81 (95% CI 0·44–1·48, p=0·50), with 176 (88%) of 200 patients in the early pramipexole group and 158 (86%) of 184 patients in the delayed pramipexole group rated as essentially unchanged from baseline. By CGI-S, the odds ratio was 0·79 (95% CI 0·40–3·30, p=0·79), with 200 (96%) of 209 patients in the early pramipexole group and 191 (96%) of 198 patients in the delayed pramipexole group essentially unchanged from baseline.

Of 160 patients in the neuroimaging substudy, 123 (77%) underwent SPECT at both baseline and 15 months. In 62 patients in the early pramipexole group and 61 patients in the delayed pramipexole group, the adjusted mean 15-month change in striatal ^123^I-FP-CIT binding was −15·1% (SE 2·1) for early and −14·6% (2·0) for delayed pramipexole, a difference of −0·5 percentage points (95% CI −5·4 to 4·4, p=0·84). In 14 (9%) patients recruited to the substudy (individuals without evidence of dopamine deficiency), baseline scans were subsequently deemed to be normal. Eight of these patients (five in the early pramipexole group and three in the delayed pramipexole group) underwent a 15-month scan. With their exclusion, the adjusted mean change was −15·5% (2·2) for early and −14·2% (2·0) for delayed pramipexole, a difference of −1·3 percentage points (95% CI −6·3 to 3·7, p=0·60).

In the post-hoc analysis of UPDRS outcome by baseline score, of the 228 patients with a baseline UPDRS total score of less than 25 as assessed by independent raters, the adjusted mean 15-month change was 1·8 (SE 0·8) points in the early and 2·8 (0·8) in the delayed pramipexole group, a difference of −1·1 (95% CI −3·0 to 0·9, p=0·28). Of the 183 patients with a baseline score of 25 or higher, the adjusted mean changes were −1·9 (1·4) in the early pramipexole group and −2·3 (1·3) in the delayed pramipexole group, a difference of 0·3 (95% CI −2·8 to 3·5, p=0·83).

A post-hoc analysis of all patients entering period 2 of the study (N=435) in which we imputed values for patients with no post-baseline data and for patients who prematurely withdrew from the study (imputed n=55), produced a difference between treatment groups in total UPDRS at 15 months of −0·44 (95% CI −2·25 to 1·37), p=0·6302.

During period 1, the frequencies of adverse events, serious adverse events, and adverse events leading to study-drug discontinuation were similar in the two treatment groups (but worsened PD was much more frequent as a reason for discontinuation in the placebo group than in the pramipexole group; figure 1), whereas severe adverse events and study-drug-related adverse events were more frequent for pramipexole than for placebo (table 4). Among serious adverse events with onset during period 1, one event (hallucinations necessitating the admission to hospital of a patient in the early pramipexole group) was judged to be study-drug-related. For patients who entered period 2, adverse events occurring at any time during the 15-month study were similar across groups (table 4). Among serious adverse events with onset during period 2, one event (orthostatic hypotension necessitating the admission to hospital of a patient in the delayed pramipexole group) was judged to be study-drug related. In both groups, the most frequently reported type of adverse event was nausea. Nine patients in the early pramipexole group and three in the delayed pramipexole group required anti-emetics, and eight patients in the early pramipexole group and two in the delayed pramipexole group withdrew as a result of nausea in period 1, and two from each group in period 2. In the early pramipexole group, 35 (13%) of 261 patients discontinued because of adverse events not related to worsening of PD, including 24 (9%) of 261 patients during period 1 and 11 (5%) of 221 patients during period 2. In the delayed pramipexole group, the frequency was 13 (6%) of 214 patients during period 2 (ie, while taking pramipexole).

**Table 4:** Adverse events

	**Early pramipexole group**	**Delayed pramipexole (placebo) group**
**Period 1**		
Number of patients treated during period 1	261	274
Any adverse events	194 (74%)	196 (72%)
Severe adverse events	34 (13%)	23 (8%)
Serious adverse events	17 (7%)	18 (7%)
Study-drug-related adverse events	113 (43%)	72 (26%)
Adverse events leading to discontinuation	25 (10%)	26 (9%)
Nausea*	54 (21%)	21 (8%)
Dizziness*	29 (11%)	24 (9%)
Somnolence*	28 (11%)	9 (3%)
Fatigue*	26 (10%)	21 (8%)
Headache*	17 (7%)	23 (8%)
Insomnia*	17 (7%)	8 (3%)
Peripheral oedema*	17 (7%)	4 (1%)
Constipation*	16 (6%)	20 (7%)
Nasopharyngitis*	16 (6%)	15 (5%)
Back pain*	14 (5%)	13 (5%)
Depression*	13 (5%)	12 (4%)
Hallucination*	13 (5%)	3 (1%)
Diarrhoea*	8 (3%)	15 (5%)
**Periods 1 and 2**		
Number of patients treated during period 2	221	214
Any adverse events	180 (81%)	179 (84%)
Severe adverse events	28 (13%)	24 (11%)
Serious adverse events	22 (10%)	17 (8%)
Study-drug-related adverse events	102 (46%)	105 (49%)
Adverse events leading to discontinuation	16 (7%)	17 (8%)
Nausea†	48 (22%)	45 (21%)
Somnolence†	30 (14%)	19 (9%)
Fatigue†	29 (13%)	34 (16%)
Dizziness†	24 (11%)	28 (13%)
Back pain†	23 (10%)	17 (8%)
Insomnia†	22 (10%)	19 (9%)
Peripheral oedema†	22 (10%)	13 (6%)
Nasopharyngitis†	18 (8%)	24 (11%)
Constipation†	17 (8%)	26 (12%)

MedDRA=Medical Dictionary for Regulatory Affairs.

* Event types (by MedDRA preferred term) reported in ≥5% of patients in either group.

† Event types (by MedDRA preferred term) reported in ≥10% of patients in either group.

By mMIDI, compulsive sexual behaviour was identified in five patients: two in the early pramipexole group (at 6 months and subsequently), and three in the delayed pramipexole group (one at baseline only, one at 12 months only, and one at 12 months and subsequently). Compulsive buying was identified in five patients, all in the early pramipexole group (one at baseline only, one at baseline and subsequently, one at 6 and 9 months only, one at 9 months and subsequently, and one at 15 months only). Compulsive gambling was not identified at any time in any patient.

## Discussion

At a maintenance dose of 1·5 mg per day in patients with early PD, at 15 months, UPDRS scores, clinical global impression ratings, quality of life ratings, and BDI score did not differ significantly between those given early pramipexole and those given delayed pramipexole. Additionally, 15-month ^123^I-FP-CIT neuroimaging findings showed a decrease in striatal dopamine-transporter binding that was equivalent in the early and delayed starters. In the first 6–9 months, the clinical efficacy of pramipexole relative to placebo in improving motor function and quality of life was confirmed at a dose limited to 1·5 mg daily, although an increase in drug-related adverse events was noted. The difference in UPDRS in period 1 was a consequence of deterioration from baseline in the placebo group; the group receiving 1·5 mg of pramipexole had returned to baseline.

The simplest explanation of the PROUD results would be that pramipexole does not have any disease-modifying action in PD, a conclusion supported by both the clinical and the neuroimaging endpoints. However, certain factors could potentially confound this interpretation. For instance, the symptomatic effect of pramipexole might have obscured a difference between the treatment groups at 15 months. This possibility is not supported by the neuroimaging data, assuming that striatal loss of dopamine-transporter binding is an accurate marker of PD-related nigrostriatal neurodegeneration and has the capacity to detect a meaningful group difference in change over 15 months. A concern is that patients with slow PD progression might have been over-represented among the PROUD participants, a consequence of the selection of patients deemed likely to tolerate a placebo phase of 6–9 months. In this case, the PROUD study design might be inherently unable to detect a disease-modifying effect that evolves over a time span exceeding 6–9 months.

The clinical results of the PROUD study can be compared with those of the Attenuation of Disease Progression with Azilect Given Once-daily (ADAGIO) study,^12^ a delayed-start trial of the monoamine oxidase B inhibitor rasagiline administered at 1 mg or 2 mg per day in early PD. Generally, the baseline characteristics of the patients recruited in these two studies were similar, although the mean UPDRS total score was 20·4 (SD 8·5) in ADAGIO, compared with 24·6 (10·9) for independent ratings or 23·9 (10·3) for study investigators' ratings in PROUD. Moreover, both trials had a target 36-week initial phase (for active treatment *vs* placebo), although ADAGIO had a second phase lasting 36 weeks whereas PROUD had a 24-week second phase. In patients who had delayed treatment, the annualised rate of progression of UPDRS total score on placebo was 6·2 points in ADAGIO, similar to the rate of 5·5 points in PROUD.

ADAGIO had three hierarchical endpoints and showed that for a dose of 1 mg a day of rasagiline, patients who received active drug early maintained a 1·7-point difference in UPDRS total-score change at week 72 compared with those in the delayed-start group. Analyses also suggested that the mean responses were not converging to one another in these two groups near the end of follow-up. Mean response at week 72 did not differ between early and delayed starters at the higher dose of 2 mg per day. A post-hoc analysis yielded results for both doses consistent with a disease-modifying effect if the analyses were restricted to patients with baseline UPDRS total scores in the highest quartile (>25·5 points), although findings for patients in the lower three quartiles then failed to attain statistical significance. This might imply that a floor effect for UPDRS rating of early PD might have masked a potential disease-modifying effect of rasagiline at the dose of 2 mg per day. In PROUD, however, the results concerning the primary outcome variable, 15-month change in UPDRS total score, were consistent in subgroups stratified by baseline UPDRS scores greater or less than 25.

Both PROUD and ADAGIO showed slope of progression for the early treated group in the first phase of the study that was slower than, and significantly different from, the slope for the untreated group. The interpretation of this result is complex. For instance, it could simply portray a gradually increasing symptomatic effect of intervention as PD progresses.

The results of PROUD have implications not only for our understanding of the use of pramipexole in early PD but also for the design of disease-modification studies in patients with PD (including the selection of patient populations and study endpoints) and for the reliance on preclinical studies and model systems to identify candidate neuroprotective drugs. PROUD confirms the clinical efficacy of pramipexole in the treatment of early PD. The timing of introduction of symptomatic therapy following diagnosis of PD remains a matter to be determined according to individual patient need. The proposal that earlier dopaminergic therapy might be associated with long-term benefits remains an interesting hypothesis,^16^ and is supported by the results of ADAGIO,^12^ the Deprenyl and Tocopherol Antioxidative Therapy of Parkinsonism study (DATATOP),^17^ and the Earlier versus Later Levodopa Therapy in PD study (ELLDOPA),^18^ but not those of PROUD (panel).

Panel **Research in context**

**Systematic review**

Extensive preclinical evidence suggests that dopamine agonists might have neuroprotective properties of relevance to Parkinson's disease (PD). On April 22, 2013, we searched in PubMed for reports of clinical trials, without restriction on language or on publication date, using the search terms “delayed-start” plus “Parkinson disease” or “Parkinson's disease”. We identified six reports: five concerning the Azilect Given Once-daily (ADAGIO)^12^, ^19^, ^20^ or TVP-1012 in Early Monotherapy for PD Outpatients (TEMPO)^21^, ^22^ studies of the monoamine-oxidase inhibitor rasagiline, and one^7^ presenting the rationale for the present study of pramipexole, a dopamine agonist. The Deprenyl and Tocopherol Antioxidative Therapy of Parkinsonism (DATATOP) study^17^ tested the ability of the monoamine-oxidase inhibitor selegiline with or without the antioxidant tocopherol to retard PD progression. Dopamine agonists have also been assessed for ability to slow the rate of progression of imaging parameters of dopamine metabolism or dopamine transporter density, compared with levodopa (Comparison of the Agonist Pramipexole versus Levodopa on Motor Complications of PD [CALM-PD]^23^, ^24^ and Ropinirole as Early Therapy versus L-dopa Positron Emission Tomography [REAL-PET]^25^ studies). All results of the published trials have been negative or inconclusive.

**Interpretation**

In a randomised delayed-start design study using 1·5 mg pramipexole during 6–9 or 15 months, we noted no difference in unified Parkinson's disease rating scale (UPDRS) total score between the early and delayed initiation groups. There was also no difference in dopamine transporter status by SPECT between the two groups at 15 months. These results indicate that pramipexole does not demonstrate an ability to slow progression of PD, as judged by UPDRS, over the 15-month period of the trial, and by contrast with the results of previous studies, does not indicate that earlier dopaminergic therapy is neuroprotective. Future clinical trials for neuroprotective drugs with symptomatic benefit will need to be designed with consideration for potential confounding factors (eg, biased PD sample, suboptimum PD stage for delaying neurodegeneration, inadequate study duration, masking by symptomatic effect, or suboptimum neuroimaging markers).

In reference to future trial design, there are several noteworthy observations. First, the proportion of patients without evidence of dopamine deficiency was smaller in PROUD than in similar studies such as ELLDOPA^18^ and the Parkinson Research Examination of CEP-1347 Trial (PRECEPT),^26^ perhaps as a consequence of the more stringent diagnostic criteria used in PROUD. Second, although patients were recruited only if thought capable of remaining untreated preferably for 9 months, about 25% failed to achieve this. The ability of the delayed-start design to address the issue of disease modification rests, partly, on the ability to minimise patient withdrawal and the resulting missing data. Excessive dropout of fast progressing patients from the placebo group might be expected to lead to a lower (ie, better) mean UPDRS score in those remaining and entering period 2. However, in PROUD, the two groups entering period 2 remained well matched, making this an unlikely explanation for the results noted. Nevertheless, future delayed-start studies need to ensure high levels of participation in both early and delayed stages and minimise dropout. Third, the standard UPDRS might not be the best assessment to identify changes in early PD, and might be limited by a floor effect.^27^

Recent insights into the causes and mechanisms of PD have provided several novel potential targets for disease modification.^28^, ^29^ Developing the most appropriate clinical trial design will be crucial to their assessment.
